# Big Data in cardiac surgery: real world and perspectives

**DOI:** 10.1186/s13019-022-02025-z

**Published:** 2022-10-29

**Authors:** Andrea Montisci, Vittorio Palmieri, Maria Teresa Vietri, Silvia Sala, Ciro Maiello, Francesco Donatelli, Claudio Napoli

**Affiliations:** 1 Division of Cardiothoracic Intensive Care, Cardiothoracic Department, ASST Spedali Civili, 25123 Brescia, Italy; 2 Department of Cardiac Surgery and Transplantation, Azienda Ospedaliera dei Colli Monaldi-Cotugno-CTO, Naples, Italy; 3 Department of Precision Medicine, University of Campania Luigi Vanvitelli, Naples, Italy; 4 Division of Anesthesiology, Intensive Care and Emergency Medicine, University of Brescia, Brescia, Italy; 5 Department of Cardiac Surgery, Istituto Clinico Sant’Ambrogio, Milan, Italy; 6 Chair of Cardiac Surgery, University of Milan, Milan, Italy; 7 Clinical Department of Internal Medicine and Specialistics, University Department of Advanced Clinical and Surgical Sciences, University of Campania Luigi Vanvitelli, Naples, Italy

**Keywords:** Big Data, Cardiac surgery, Artificial intelligence, Machine learning, Coronary revascularization, Valvular heart diseases, Heart failure, Left ventricular assist devices

## Abstract

Big Data, and the derived analysis techniques, such as artificial intelligence and machine learning, have been considered a revolution in the modern practice of medicine. Big Data comes from multiple sources, encompassing electronic health records, clinical studies, imaging data, registries, administrative databases, patient-reported outcomes and OMICS profiles. The main objective of such analyses is to unveil hidden associations and patterns. In cardiac surgery, the main targets for the use of Big Data are the construction of predictive models to recognize patterns or associations better representing the individual risk or prognosis compared to classical surgical risk scores. The results of these studies contributed to kindle the interest for personalized medicine and contributed to recognize the limitations of randomized controlled trials in representing the real world. However, the main sources of evidence for guidelines and recommendations remain RCTs and meta-analysis. The extent of the revolution of Big Data and new analytical models in cardiac surgery is yet to be determined.

## Introduction

The combination of data coming from multiple sources, and constituting databases, with significant possibility of integration and complex aggregation and discriminant analyses, defines the so-called Big Data, which intrinsically refers to extensive datasets, widely informative for a large number and variety of persons.

In fact, large and rapidly increasing amount of data (*Volume*), their multiple sources (i.e., clinical studies, registries, small database, administrative database, patient-reported outcomes, genomic profiles and environmental parameters) (*Variety*), the rapid data accumulation (*Velocity*) and their ability to truly represent a specific context (*Veracity*) characterize Big Data [1].

Analyses of these data may unveil patterns, trends and associations, and define reference models in aggregations of persons [2]. As data digitalization and information technology (IT) are spreading and improving performance [3–5], the use of Big Data is steeply increasing, becoming progressively a reference for many typical processes in medicine, such as the identification of the appropriate therapeutic choice by tailoring therapeutic options, the evaluation of short and mid-term, procedure-related or unrelated, risks of adverse events and the definition of the prognosis. To such an extent, Big Data may generate the basis for precision medicine, as factors impacting event occurrence is progressively available for the single subject and may improve effectiveness of cardiovascular therapies [6].

**Fig. 1:**
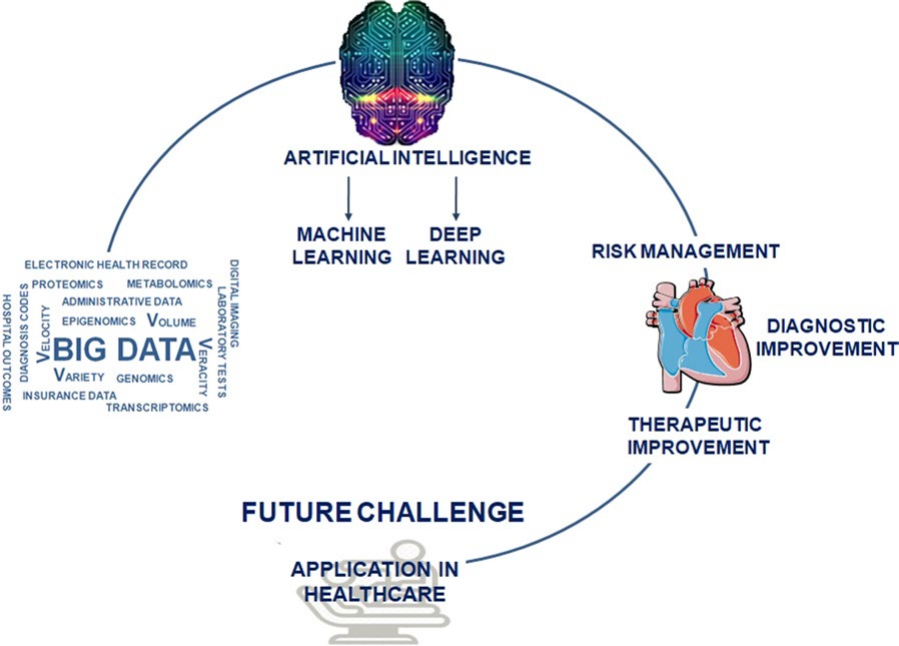
Big Data elaboration allows an improvement of risk management, and diagnostic or therapeutic strategies in cardiac surgery. The future challenge will be the practical application in healthcare

The place of Big Data is far from being well determined. Figure 1 offers a graphical synthesis of the present and future of Big Data.

## Big Data: sources and analysis

The key points of the application of Big Data are the clinical usefulness and the balance between costs of sophisticated data analyses and the *expected* and *real* benefits (i) to patients, in terms of quality of care, outcomes and risk prediction; (ii) to operators, in terms of quality and security of processes, from the diagnosis to the choice of therapeutic options, in a perspective of resources saving.

Regarding the sources, Electronic Health Record (EHR) is the main source of Big Data. Administrative data, which are commonly employed for billing purposes in fee-for-service health systems, may turn helpful for a large spectrum of analytic goals, and may fuel risk modeling for clinical and economical purposes [7]. Several approaches are used to enable data aggregation from EHR and to facilitate their contribution to Big Data. The most relevant limitations of the use of these databases are the risk of misclassification and the impact of missing data [8].

Digital imaging significantly contributes to Big Data generation. Today, almost all medical images are stored in pixels or voxels [9], which can be processed by software aiming at improving data quality and diagnostic accuracy [10].

Finally, OMICS datasets, or genome, proteomic, transcriptomic, epigenomic, and metabolomic data, are readily available in digital and structured forms, allowing the recognition of patterns useful for grouping procedures; they are additional important sources for Big Data [11].

In cardiovascular medicine, the contribution of OMICS is enormous and hold a very high potential for Big Data generation and subsequent analysis. A valid example of the OMICS data analysis is that it is able to provide information on myocardial molecular profiles of cardiac surgical patients [12–14]. The variants identification by OMICS techniques allows performing association studies, which may turn useful to risk stratification and outcome prediction in the context of precision medicine [15–19], using appropriate systems for analyses [20–22].

When it comes to analyses of Big Data, classic statistics is limited. Independent storage systems, immediate access and relational databases procedures are crucial for Big Data analyses. Artificial intelligence (AI) is of a particular usefulness, conceived as computer and mathematical concept allowing “machines” to execute learning, problem-solving, patterns recognition, reasoning and planning, in a way that resembles “human thinking”, therefore dealing with uncertainty, projection, and production that are well beyond extrapolating regression models validated in subsets of the general population, called validation samples, to approximate prediction in the general population.

The ability of such a technology to process decisions independently, recognize errors and readjust the decision or prediction models and processes, are the main characteristics of the AI, based on machine learning (ML) and deep learning (DL).

ML is the study of a mathematical algorithm model from sample data which in turn is used to generate predictions or decisions. ML algorithms can be supervised, unsupervised and reinforced [23]. DL is composed of artificial neural networks, with representation learning, to mimic human cognition. DL could be a module to automate predictive analysis, from which data is deduced in a non-linear way. The advantage of a non-linear interpretation is the better ability to identify and interpret more complex characteristics [24] and therefore is linked to a hierarchy of increasing complexity and abstraction [25]. DL is used for image evaluation, such as cardiac magnetic resonance scans; this requires adequate skills and systems [26, 27].

Logistic regression (LR) is a classic classification algorithm that makes a linear combination of input variables and uses the sigmoid function to output a probability.

Neurons in artificial neural networks (ANN) make a linear combination of the output value from the upper layers’ neurons, pass it through sigmoid functions, and finally output a value to the next neurons [28].

The use of predictive models that evaluate the influence of covariates, in the prediction of the results, allows it to identify the patients for whom the intervention will be successful. However, in analyzing non-randomized diagnostic or therapeutic strategies, it is possible to compare non-similar groups, exposing patients to subsequent complications [2].

## The present of Big Data in cardiac surgery and the road ahead

While procedures safety and outcomes in cardiac surgery have improved over the years in the majority of elective procedures, cardiac surgery is facing patients with increased complexity due to improved survival owing to refined cardiological, pneumological and oncological therapies. Changes in such a clinical context require rethinking clinical risk assessment and management as well as redefinition of optimal timing for surgical options.

So far, cardiac surgery has relied on logistic models to estimate the risk of events, including mortality associated with cardiac surgery, as essential components of routine clinical management of cardiac surgical patients. The EUROscore II and the Society of Thoracic Score (STS) scores are the logistic models for cardiac surgery-related risk stratification most commonly employed. Nevertheless, the prediction of those estimates is debated, especially in subsets of patients [29]. AI applied to Big Data has the potential to change the paradigm, from a theoretical and average risk prediction to simulations in single patients, weighting tailored therapeutic options and managing risks, and finally employing sustainable options to improve outcomes.

This is the most appealing application of the new concept of the meta-verse, an aggregate of Big Data, information technologies and AI converging to generate novel approaches to handle reality by navigating virtual near-future in clinical contexts, hopefully yielding time saving, less errors, more precision, variability due to operators, minimize costs and human effort while prolonging life with reasonable quality.

To such an extent, Big Data and AI have been applied in seminal studies in cardiac surgery in the field of myocardial revascularization, valvular heart diseases and end-stage heart failure (Table 1). Table 2 shows the ongoing trials focused on the application of Big Data-derived analysis in cardiac surgery.

**Table 1:** Artificial intelligence in cardiac surgery and cardiovascular diseases

Author	Title	Year	AI Model	Conclusions
Diller et al. [66]	Machine learning algorithms estimating prognosis and guiding therapy in adult congenital heart disease: Data from a single tertiary centre including 10 019 patients.	2019	Deep Learning	Prognostication and therapeutic guidance in patients with adult congenital heart disease (ACHD) or pulmonary hypertension
Diller et al. [67]	Utility of machine learning algorithms in assessing patients with a systemic right ventricle.	2018	Deep Learning. Convolutional neural networks	Recognizing transposition of the great arteries (TGA) after atrial switch procedure or congenitally corrected TGA (ccTGA) based on routine transthoracic echocardiograms. Delineation and segmentation of the systemic ventricle
Olive et al. [68]	Current monitoring and innovative predictive modeling to improve care in the pediatric cardiac intensive care unit.	2018	AI and machine learning	Predictive models created by AI and ML may lead to earlier detection of patients at risk for clinical decompensation, improving care for critically ill pediatric cardiac patients.
Ruiz-Fernández et al. [69]	Aid decision algorithms to estimate the risk in congenital heart surgery.	2017	Multilayer perceptron, self-organizing map, radial basis function networks and decision trees	Feasibility of development of CDSSs using AI algorithms. Such system would help to forecast the level of risk related to a congenital heart disease surgery.
Zhong et al. [70]	Machine learning prediction models for prognosis of critically ill patients after open-heart surgery	2021	Extreme gradient boosting, random forest, artificial neural network, and logistic regression	Model to predict 30-days mortality and complications (i.e.: septic shock, thrombocytopenia and liver disfunction) after open-heart surgery.
Meyer et al. [71]	Machine learning for real-time prediction of complications in critical care: A retrospective study	2018	deep learning methods (recurrent neural networks)	Predict severe complications (i.e.: mortality, renal failure with a need for renal replacement therapy, and postoperative bleeding leading to operative revision) during critical care in real time after cardiothoracic surgery.
Lei et al. [72]	Using Machine Learning to Predict Acute Kidney Injury After Aortic Arch Surgery	2020	Machine Learning: logistic regression model, support vector machine, random forest, and gradient boosting	Machine learning methods were found to predict AKI after aortic arch surgery significantly better than traditional logistic regression.
Tseng et al. [73]	Prediction of the development of acute kidney injury following cardiac surgery by machine learning	2020	Logistic regression, support vector machine (SVM), random forest (RF), extreme gradient boosting (XGboost), and ensemble (RF + XGboost)	AI methods predict cardiac surgery-associated acute kidney injury, which determines risks following cardiac surgery, enabling the optimization of postoperative treatment strategies.
Lee et al. [74]	Derivation and Validation of Machine Learning Approaches to Predict Acute Kidney Injury after Cardiac Surgery	2018	ML: decision tree, random forest, extreme gradient boosting, support vector machine, neural network classifier, and deep learning.	Using AI an Internet-based risk estimator was developed to estimate the risk of AKI at the end of surgery.
Kilic et al. [75]	Performance of a machine learning algorithm in predicting outcomes of aortic valve replacement.	2020	Extreme gradient boosting (XGBoost)	Predicting outcomes of surgical aortic valve replacement.
Wojnarski et al. [76]	Machine-learning phenotypic classification of bicuspid aortopathy	2018	Random forest analysis	Three distinct phenotypes of bicuspid valve-associated aortopathy were identified using machine-learning methodology.
Baskaran et al. [77]	Machine learning insight into the role of imaging and clinical variables for the prediction of obstructive coronary artery disease and revascularization: An exploratory analysis of the CONSERVE study	2020	ML: extreme gradient boosting (XGBoost)	For obstructive CAD, the ML model outperformed CAD consortium clinical score (CAD2). BMI is an important variable, although currently not included in most scores. In this ML model, imaging variables were most associated with revascularization
Cikes et al. [78]	Machine learning-based pheno-grouping in heart failure to identify responders to cardiac resynchronization therapy	2018	Unsupervised multiple kernel learning algorithm (MKL)	Integrating clinical parameters and full heart cycle imaging data, unsupervised ML can provide a clinically meaningful classification of a phenotypically heterogeneous HF cohort and might aid in optimizing specific therapies.
Ambale-Venkatesh et al. [79]	Cardiovascular Event Prediction by Machine Learning: The Multi-Ethnic Study of Atherosclerosis	2017	Random survival forest (RF) alone or in combination with other statistical approaches.	Machine learning in conjunction with deep phenotyping improves prediction accuracy in cardiovascular event prediction in an initially asymptomatic population.
Ayers et al. [80]	Using machine learning to improve survival prediction after heart transplantation	2021	Deep neural network, logistic regression, AdaBoost, and random forest	ML techniques can improve risk prediction in OHT compared to traditional approaches. This may have important implications in patient selection, programmatic evaluation, allocation policy, and patient counseling and prognostication.

**Table 2:** Ongoing trials on the application of big data and derived analysis techniques to cardiac surgery

Title	Location	Status	Description
Effects of AI Assisted Follow-up Strategy on Secondary Prevention in CABG Patients ClinicalTrials.gov Identifier: NCT04636996	Cardiovascular Institute and Fuwai Hospital, Beijing, Beijing, China	Not yet recruiting	Assess if AI assisted follow-up strategy will improve secondary prevention in CABG patients
Artificial Intelligence Guided Patient Selection for Atrial Fibrillation Catheter Ablation: Randomized Clinical Trial (AI-PAFA Trial) ClinicalTrials.gov Identifier: NCT04997824	Severance Hospital, Yonsei University Health System Seoul, Korea, Republic of	Not yet recruiting	Prediction of AF catheter ablation (AFCA) efficacy using artificial intelligence (AI)
Effect of Artificial Intelligence on Nutritional Status of Children Post Cardiac Surgery ClinicalTrials.gov Identifier: NCT04782635	Armed Forces Institute of Cardiology and National Institute of Heart Disease Rawalpindi, Punjab, Pakistan Maryam Zahid Rawalpindi, Punjab, Pakistan	Completed	Assess the effect of artificial intelligence on nutritional status of children post cardiac surgery in comparison to usual care group
Cloud-based ECG Monitoring and Healthcare Model Building on the Population With Coronary Artery Revascularization ClinicalTrials.gov Identifier: NCT04485143		Not yet recruiting	All subjects tracked the occurrence of adverse medical events within one year after discharge from the hospital. Based on the home-based remote personal care model for patients with CABG, a risk prediction model for heart failure and vascular restenosis was established to effectively reduce medical treatment, adverse events, and medical expenditure
Machine Learning Predict Acute Kidney Injury in Patients Following Cardiac Surgery ClinicalTrials.gov Identifier: NCT04966598	Chinese PLA General hospital Beijing, Beijing, China	Completed	Several prediction models based on machine learning technique are developed to allow early identification of patients who at the high risk of unfavorable kidney outcomes
Machine Learning-Based Prediction of Major Perioperative Allogeneic Blood Requirements in Cardiac Surgery (PREMATRICS) ClinicalTrials.gov Identifier: NCT04856618	Kepler University Hospital Linz, Upper Austria, Austria	Recruiting	If an accurate prediction model based on a few features could be created and those patients particularly at risk of massive transfusion of allogeneic blood could be identified, it would subsequently be possible to develop an adapted clinical pathway that would allow patient care to be improved and individualized interventions adapted to the situation to be implemented
Machine Learning-Based Risk Profile Classification of Patients Undergoing Elective Heart Valve Surgery ClinicalTrials.gov Identifier: NCT03724123		Completed	The investigators investigate the benefit of modern machine learning methods in personalized risk prediction in patients undergoing elective heart valve surgery
Remote Monitoring to Improve Physician Monitoring, Patient Satisfaction, and Predict Readmissions Following Surgery ClinicalTrials.gov Identifier: NCT03800329	Mayo Clinic in Rochester Rochester, Minnesota, United States	Completed	Measure data collected via machine learning algorithms to predict readmission following cardiac surgery

We interrogated ClinicalTrial.gov and EUDRACT databases with the following keywords: Coronary Heart Disease AND Artificial Intelligence, cardiac surgery AND Artificial Intelligence, cardiac surgery AND machine learning, cardiac surgery AND big data. A total of 811 studies were found on ClinicalTrial.gov and a total of 10 on EUDRACT; we selected the most appropriated for our purposes

*CABG* coronary artery bypass grafting, *AI* artificial intelligence, *AF* atrial fibrillation

### Myocardial revascularization

In the field of surgical myocardial revascularization, structured data from EHR managed by the Society of Thoracic Surgeons, the American College of Cardiology (National Cardiovascular Data Registry), and the American Heart Association, represent a significant source for data analysis. Clinical records, and data from imaging, analyzed and interpreted using AI approaches, may integrate original approaches based on clinical registries and regression models (e.g.: neural networks) [9, 30].

One of the major challenging research tasks, to date, is to evaluate whether percutaneous coronary interventions (PCI) are superior over coronary artery bypass surgery (CABG) in specific clinical contexts. Randomized trials (RCTs) on myocardial revascularization have been used to answer this question, and to develop a tool for the identification of patients who may benefit from one therapeutic option over the other or a combination of both (hybrid revascularization), with minimized clinical risk and expenditure. Although RCTs are very effective to control treatment selection bias, they are low-performing in evaluating subgroups and they suffer from inappropriate statistical power in subset and possible *post-hoc* biases. Observational, nonrandomized data gathered from registries and large multi-sources databases, may be closer to real-world representing a very large majority of patients, and therefore may work better with single patients using AI based analytic modalities. In contrast, those sources of data may be affected by lower data control and potential biases in the outcome definition and assignment [31]. Weintraub and colleagues [32, 33] compared the effectiveness of different myocardial revascularization strategies by linking the American College of Cardiology Foundation (ACCF) National Cardiovascular Data Registry and the Society of Thoracic Surgery (STS) Adult Cardiac Surgery Databases, to data from claims from the Centers of Medicare and Medicaid. They demonstrated that the real-world mortality was not significantly different at 1-year from that anticipated by commonly used scores, while long term survival was higher in patients receiving CABG as compared with patients who underwent PCI. The method of analyses of these data required the use of probabilistic matching to identify patients throughout databases, adjusting for clinical covariates with the use of inverse probability weighting, and correction of residual confounding by means of a sensitivity analysis.

A valid example of cost-effective applicability of AI to Big Data in the field of myocardial revascularization is the ASCERT study (ACCF and STS Database Collaboration on the Comparative Effectiveness of Revascularization Strategies) [34], where two revascularization strategies (PCI versus CABG) were evaluated in patients suffering from stable ischemic heart disease. linking large converging databases, both clinical and administrative, to obtain data from 86,244 patients for CABG and 103,549 patients for PCI. Those figures are much larger than any proposed by RCTs. Interestingly, the authors found that patients undergoing CABG had better outcomes than those undergoing PCI, but at the expense of higher costs, allowing the calculation of the indicator of the incremental cost-effectiveness ratio expressed as cost per quality-adjusted life-year gained.

The role of some patients-related characteristics in determining outcomes in myocardial revascularization strategies are highlighted in the large study by Hlatkyet al. [35], where authors demonstrated lower long-term mortality with CABG rather than with PCI, in an unselected group of patients extracted by the general population of those undergoing those procedures, with outcomes substantially modified by factors such as diabetes, smoking habit, heart failure, and peripheral artery disease.

The fundamental study of Weintraub et al., by linking the STS database to that of the Centers for Medicare and Medicaid Services, showed that in stable patients, older than 65 years with multivessel coronary artery disease, CABG offers an advantage in terms of long-term survival.

While RCTs remain the only accepted evidence-based information gathered in medical guidelines, studies based on Big Data and AI added knowledge on the comparative effectiveness of the two therapeutic strategies. The place of Big Data analysis is therefore yet to be precisely determined.

### Valvular heart diseases and cardiac imaging

The identification of significant valvular diseases has the potential to clarify the etiology, and/or reveal a consequence of ventricular failure, with or without dilatation, which may contribute to define the prognosis and to identify elements acting as triggers for worsening heart failure and hence prognosis. Echocardiography is used widely for assessment of cardiac structure and function, with diagnostic accuracy and reliability depending on the operators’ skill, experience and expertise. In contexts such as high volume in busy environment, and emergency, machines enriched with a technology that analyses live imaging in real-time, continuously comparing it with a pool of reference images, may help optimizing the imaging protocol and identifying diseases or patterns of abnormalities, detecting trends over time and evaluating the stability of specific measurements. Those are intriguing perspectives that may be associated with the application of AI in the field of echocardiography. For instance, in mitral valvular regurgitation, recognition of increasing severity of the valvular insufficiency, ventricular dilatation and reduction in chamber shortening, atrial dilatation, as well as the worsening myocardial function assessed by means of semiautomated, relatively load-independent parameters, may parallelly run with the detection of clinical changes and even anticipate overt changes in symptoms and signs of heart failure (HF).

In echocardiography, automated views recognition and structures identification may be considered an initial step toward semi-assisted diagnostic studies. To such an extent, AI-based technology is a key element, as convolutional neural networks may be employed to identify key reference points on images, and then feature specific for diagnostic patterns. Identification of normal patterns, deviations from normal patterns or specific pathologies has been possible in experimental studies in more than 9 cases in 10 evaluated cases [36] in algorithm-based supervised machine learning [37]. A further step in AI-assisted echocardiography may be the identification of deviation from physiology or overt pathological conditions, such as left ventricular hypertrophy, hypertrophic cardiomyopathy [38, 39], ventricular dilatation and reduced chamber and myocardial function [40]. With 2-D speckle-tracking technology, an accurate semi-automated volume and systolic function quantification may be run bed-side and take a few minutes or seconds [41, 42]. In the context of valvular disease associated with ventricular dysfunction, an important role is played by quantification of contractility reserve, which impacts prognosis. Wall motion quantification is relevant to such an extent, with AI operated diagnostic modality pushing accuracy of wall motion quantification as high as 85% [43, 44], helping in the case of evaluation of ventricular contractility reserve and aiding in decision making on the best valvular disease management.

Assessment of mitral valve regurgitation severity may be aided by automated processes based on deep learning machines [45, 46]. With regard to aortic valve disease, identification of trends over time in ascending aorta and aortic root dimensions, ventricular dimensions and shortening, relies on reproducibility of imaging in specific views, and may provide important information impacting preclinical and, timely, clinical decision making. Moreover, aortic annulus sizing represents an important target of quantification, as to date transcatheter procedures for aortic valve replacement are increasing steeply [47].

Beyond echocardiography, the potential revolution of AI may be even more applicable and profound in more standardized diagnostic processes such those applicable to nuclear medicine, computed tomography and magnetic resonance imaging, which may suffer much less variability between-subjects due to body size [48, 49].

### HF and mechanical circulatory support: selection of patients, prediction of adverse events and technology development

Heart transplantation (HTx) is the gold standard therapy in advanced HF, defined as persistence of symptoms and significant personal physical limitations despite optimized pharmacological and standard nonpharmacological therapy, associated with recurrent hospitalizations and need for escalation therapy including inotropes [50]. However, the number of organs available does not match the number of patients in need for organs as they slide toward a deterioration of the clinical conditions and require a timely intervention. Left Ventricular assist devices (LVAD) may help as bridge to HTx, or to candidacy or decision, or even as destination therapy in subjects with advanced or terminal heart failure [51, 52]. Those patients, suffering from end-stage HF, present with unique challenges: frailty [53], end-organ damage, risk for acute decompensations, and high mortality at short-term. Events as cardiogenic shock carries worsened prognosis. The therapeutic strategy shows multiple options, with multiple devices that can be employed in different phases of the clinical course [54].

Because of the high risk of surgery and the patients’ characteristics, the prediction of peri-procedural adverse events and of the long-term complications are critical issues when planning LVAD implantation, impacting benefit and quality of life per costs [55], and healthcare sustainability. Data from registries and discriminant statistics are commonly used to identify potentially life-threatening conditions impacting prognosis and hospital stay duration after LVAD implantation [56].

However, the classic way for risk-prediction estimation is based on statistical methods that implies a proportional and statistically significant participation of several variables in a context where hazard is not proportional over time [57]. On a different approach, AI and Big Data may simulate the effects of decisions, and their interactions with an uncertain environment [58] and facilitate not only the prediction of specific pre-defined events.

Prediction of complications after LVAD implantation is relevant to sustainability of LVAD procedures. AI has been involved to recognize drive-line infections using photographic database as background source [59] and the identification of clusters of variables that predict right ventricular failure, bleeding, infection and pump failure due to pump thrombosis [60].

While statistically-based risk models have proven suboptimal ability to predict mortality risk in LVAD, by use of Interagency Registry for Mechanically Assisted Circulatory Support (INTERMACS), data from 2006 to 2016, for a total of 16,120 patients included, and bootstrapping with 1000 replications in the testing set, improved 90-day discrimination from 0.707 [0.683–0.730] to 0.740 [0.717–0.762] and 1-year mortality from 0.691 [0.673–0.710] to 0.714 [0.695–0.734] (all *p* < 0.001). The net reclassification rate was up to 49% for 90-day mortality and 37% for 1-year mortality. The findings supported the concept that ML may increase the performance of a risk model for durable LVAD mortality compared to logistic regression-based algorithm [60]. Because continuous blood flow from LVAD is associated with increased risk of complications, as gastrointestinal bleeding, continuous pump speed generating flow is modulated to generate pulsatile flow. More importantly, pump speed of the LVAD may be controlled to assist left ventricles during a single beat to optimize systolic, versus diastolic assistance [61]. Diastolic versus systolic modulation of the pump speed may impact on flow pulsatility and diastolic assistance to reduce external myocardial work [62]. These mathematical models are limited in their applicability because the need for pressure feedback signals from the cardiovascular system require suitable integrated long-term pressure sensors. To date, novel AI based controllers, real-time deep convolutional neural network-based, are tested to estimate left ventricular preload using LVAD flow analyses and a sensorless adaptive control system, trained and evaluated through a number of cross validation settings and physiologic situations in different patient and different conditions, resulting in accurate pre-load evaluation (root mean squared error of 0.84 mmHg, reproducibility coefficient of 1.56 mmHg, coefficient of variation of 14.44%, and bias of 0.29 mmHg for the testing dataset) [63]. The system was able to use LVAD data to measure preload and prevent ventricular suction and pulmonary congestion [64].

## Conclusions

After several years of intense research yielding a great number of scientific publications, a major gap exists between practical application of AI applied to Big Data and RCTs to guide practice in real work, in large part because AI and Big Data are yet to become controlled research tools on a large scale. Data quality control, missing data, privacy and potential conflicts of interests in a variety of stakeholders, costs of the technology required, and the need for high-performing information technology are still barriers for a routine and wide use of Big Data in the field of cardiac surgery research and clinical process.

Nevertheless, in the context of an enormous resource re-allocation due to the COVID-19 pandemic, that reduced significantly the research output in other fields of medicine, Big Data and AI may turn to be relevant tools.

The role of hypothesis generation in Big Data science is without doubt, but it should be considered as a complementary mean to obtain evidence [65].

However, the crucial match on usefulness of Big Data and AI in the near future is also played in the side of productivity, simulation, augmented reality aiding diagnostic and clinical decision making, communication with patients and generating precision medicine. These features have the potential to go well beyond the context of knowledge generated from RCTs to prove or unconfirm specific hypotheses, by using strict enrollment criteria to make homogenous the population. Hence, we all need to be familiar with those concepts and tool for the future, which is not that far away from now.

## Data Availability

Not applicable.
